# The Book World of Medicine and Science

**Published:** 1893-08-05

**Authors:** 


### THE BOOK WORLD OF MEDICINE AND SCIENCE.

### BOOK REVIEWS.

THE PHYSIOLOGIST'S NOTE-BOOK. A Summary of the Present State of Physiological Science, for the use of Students. By ALEX. HILL, M.A., M.D., Master of Downing College, Cambridge. (Charles Griffin and Co., Limited, London. 1893.) 200 pp., 36 plates.

THE volume before us claims attention, bearing as it does, the hall-mark of the Cambridge School of Physiology, and the name of one of the most successful of its teachers. It belongs to a class of work which appeals especially to the student, inasmuch as it helps him to systematise his studies, to codify his knowledge, and from time to time to take, as it were, a bird's eye view of his subject, or of any section of it. To say this is not equivalent to classifying the work among the cram-books with which it has nothing in common. The evolution of our knowledge of each physiological fact is succinctly yet clearly stated, and on questions where absolute facts have not yet been reached, the present state of our information, and the means by which it has been attained are indicated, as well as a brief statement of the difficulties and doubts which surround it. The student is asked to take nothing for granted, the "pros" and "cons" are always forthcoming. Nothing could be clearer, for example, than the paragraph on the causes of the first sound of the heart. The various theories are successively stated, each criticised in a few words, and by a somewhat subtle arrangement of the type on the page an idea of the value of each statement is conveyed to the reader. With such a work as this in his hands the slavish note-taking on which the student wastes so much of his energy will be no longer necessary. The running bibliography of British literature which is given at the foot of each page is a feature of the work which is to be commended, and it will doubtless be highly appreciated by the advanced student. The text is illustrated by a large number of excellent figures, the majority of which are new, and all of which have the merit of showing exactly what is wanted, and neither more nor less. They are diagrams rather than pictures. A glance at Figs. 65, 66, and 67 will demonstrate what pages of text would fail to convey regarding errors in sensory judgments. From Fig. 81 the student will learn the complicated cerebral and muscular mechanism he sets into action when he attempts to take down to dictation a physiological lecture, and a comparison of his own note-book with Dr. Hill's work will show how futile have been his efforts. To the students of physiology in the Cambridge school this book will prove invaluable, and we anticipate its early popularity in other places.

WHAT TO DO IN CASES OF POISONING. By WILLIAM MURRELL, M.D., F.R.C.P. Price 3s. 6d. (Published by Lewis and Co., 136, Gower Street.)

The seventh edition of this excellent little book will receive a cordial welcome. It contains valuable additions and instructions as to new drugs, &c. Cases of chronic poisoning are now dealt with in a separate section, and the book, as the author says in the preface, "is essentially a

THE BOOK WORLD OF MEDICINE AND SCIENCE.—(*continued*).

pocket guide, and has no higher aim than to give in the plainest possible language directions for the use of antidotes in cases of poisoning." Many useful hints as to the diagnosis and the legal aspects of poisoning are put before the reader, and it seems implied that the latter is at any rate a student, if not a fully-qualified practitioner, but this book is too interesting for its perusal to be confined to only one kind of reader. Not only will nurses discover most useful hints therein, but no one can fail to benefit by such information as that as to soothing syrups and other similar preparations by which 15,000 children are killed every year. The very fact that the book is one likely to attract many unprofessional readers makes us regret that such very vague directions as the following occur in more than one place, "In apparently hopeless cases two or three violent blows on the chest delivered in quick succession may restore the heart's action.' Of course the qualified practitioner, the intelligent student, and the trained nurse can carry out this order safely, but an ignorant attendant might inflict such injury on the person suffering from poison as might add considerable complications to his condition. The addition of a few words to the text would obviate the risk of "violent blows" being dealt with more zeal than discretion by the frightened and unskilled spectator. Practical suggestions as to the systematic treatment of habitual opium eaters are given on most common-sense lines, and we must all agree that "the cooking should be good, and, above all, varied. You will never cure a man of anything unless you feed him well and properly."

